# *Mamu*SNP: A Resource for Rhesus Macaque (*Macaca mulatta*) Genomics

**DOI:** 10.1371/journal.pone.0000438

**Published:** 2007-05-09

**Authors:** Ripan S. Malhi, Brad Sickler, Dawei Lin, Jessica Satkoski, Raul Y. Tito, Debbie George, Sreetharan Kanthaswamy, David Glenn Smith

**Affiliations:** 1 Department of Anthropology, University of Illinois Urbana-Champaign, Urbana, Illinois, United States of America; 2 Institute of Genomic Biology, University of Illinois, Urbana-Champaign, Urbana, Illinois, United States of America; 3 Bioinformatics Core, Genome Center, University of California Davis, Davis, California, United States of America; 4 Department of Anthropology, University of California, Davis, Davis, California, United States of America; 5 California National Primate Research Center, University of California, Davis, Davis, California, United States of America; Ecole Normale Supérieure de Lyon, France

## Abstract

We developed a novel method for identifying SNPs widely distributed throughout the coding and non-coding regions of a genome. The method uses large-scale parallel pyrosequencing technology in combination with bioinformatics tools. We used this method to generate approximately 23,000 candidate SNPs throughout the *Macaca mulatta* genome. We estimate that over 60% of the SNPs will be of high frequency and useful for mapping QTLs, genetic management, and studies of individual relatedness, whereas other less frequent SNPs may be useful as population specific markers for ancestry identification. We have created a web resource called *Mamu*SNP to view the SNPs and associated information online. This resource will also be useful for researchers using a wide variety of *Macaca* species in their research.

## Introduction

Rhesus macaques (*Macaca mulatta*) are a widely used and valuable model for biomedical research and understanding the etiology of human diseases. With the completion of a draft sequence of the rhesus macaque genome [1], the ability to identify genetic polymorphisms throughout the entire genome will vastly improve the utility of rhesus macaques for investigating the heritable components of complex diseases. In particular, the identification of SNPs (Single Nucleotide Polymorphisms) will contribute to a high-density linkage map that can be used to perform QTL (Quantitative Trait Loci) and association studies to identify genes that contribute to a phenotype of interest. Research to begin finding diagnostic SNPs within or near genes in the *M. mulatta* genome has already begun [2], [3]. In addition SNPs are useful markers for genetic management in captive colonies because they are widely distributed and abundant in the genome and are amenable to automated high-throughput analyses.

Large-scale parallel pyrosequencing technology from 454 Life Science™ is a new method that can quickly obtain hundreds of thousands of short (approximately 100bp) DNA sequences. Due to the nature of the sequencing method, these sequences are distributed randomly across the entire genome. This wide range of coverage makes it advantageous to discover unlinked common SNPs in coding and non-coding regions by screening one or only a few animals. This method of SNP identification is unique because it can be used to identify SNPs distant from any genes. Therefore, this method is useful for identifying regulatory regions located in genomic deserts far from a gene, but which still contribute to variation in a phenotype (e.g. [4]).

Here we use large-scale parallel pyrosequencing from 454 Life Science™ to generate approximately 23,000 candidate SNPs throughout the rhesus macaque genome; the candidate SNPs are distributed across all 20 autosomes and the X chromosome. Each candidate SNP is mapped to an exact location on the current draft of the genome (07/06) and flanking DNA sequence for each SNP is provided so that primers can be easily designed to assay the SNP. Over 1,500 SNPs were sequenced at least twice through the pyosequencing process and a subset of these SNPs have been confirmed via Sanger sequencing in a panel of animals from geographically diverse origins. All candidate SNPs and associated information can be accessed at the website http://mamusnp.ucdavis.edu. Updated SNP frequencies in populations as well as additional verified SNPs will be routinely uploaded to the website.

Cynomolgus macaques (also known as longtail or crab-eating macaques), *Macaca fascicularis,* are becoming an important non-human primate model and may soon outnumber rhesus macaques in biomedical research. Doxiadis et al. [5] has recently shown a relatively high level of allele sharing at class II MHC loci between rhesus and cynomolgus macaques. In addition, a high level of allele sharing has also been reported at STR loci between the two species (Satkoski pers com). Therefore many of the SNPs identified in rhesus macaques and reported on the *Mamu*SNP website may also be polymorphic in cynomolgus macaques and other closely related primates. Although these SNPs would exhibit a significant ascertainment bias in cynomolgus macaques, they may still be useful as markers for QTL analyses.

## Results

**Figure 1:**
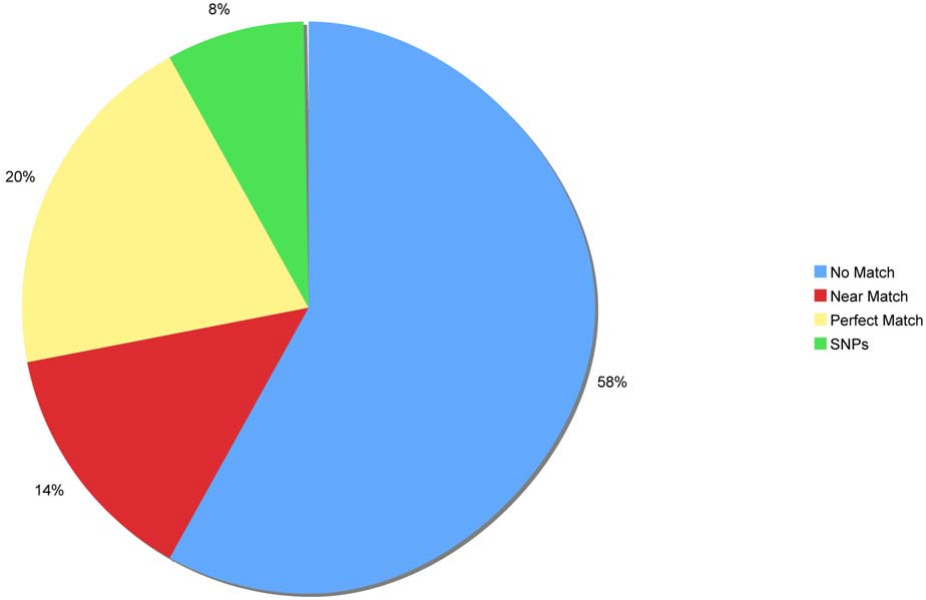
Proportion of 454 pyrosequencing DNA fragments that exhibited SNPs. No match indicates a DNA fragment not from *M. mulatta*. A near match indicates a DNA fragment with at least a 98% match to *M. mulatta* but unsuitable for SNP detection. A perfect match indicates a DNA fragment with a 100% match with *M. mulatta*. A SNP indicates a DNA fragment with a near perfect match with *M. mulatta* and suitable sequence for SNP detection.

**Figure 2:**
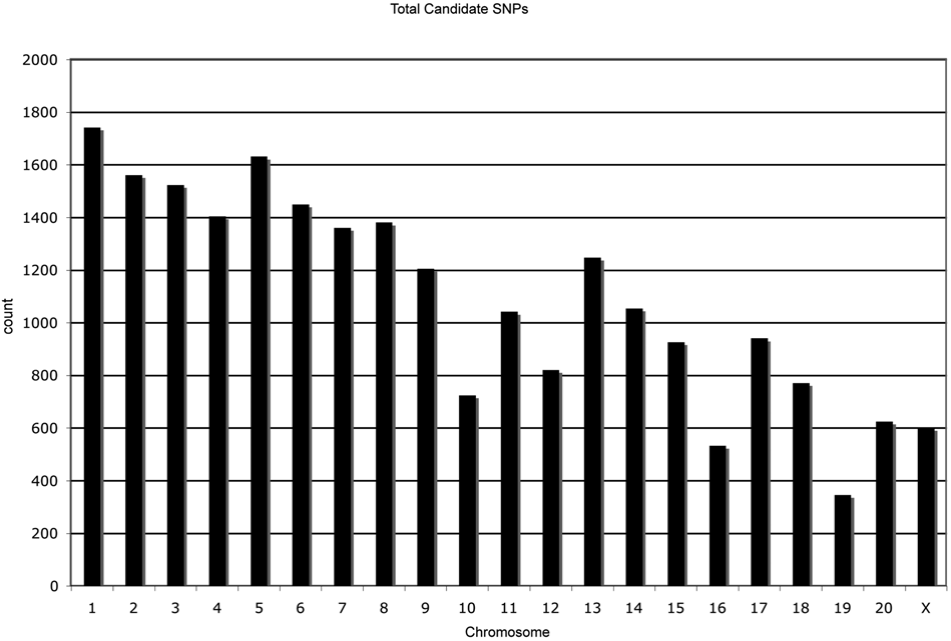
Distribution of Candidate SNPs across genome.

**Figure 3:**
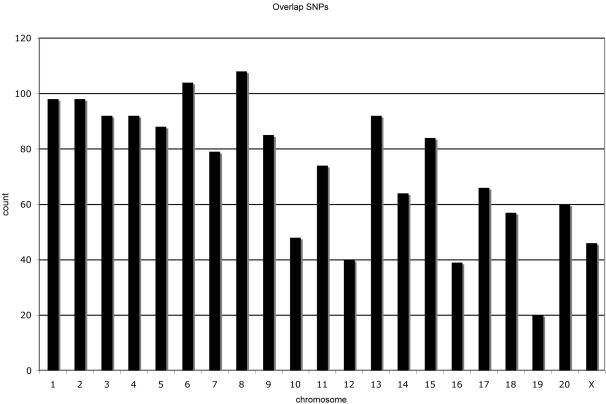
Distribution of overlap SNPs across the genome.

The average read length received from 454 Life Science™ was 103.92 bases. The DNA sequence was of high quality except within regions of homopolymer tracts, a known limitation of pyrosequencing. We obtained 339,967 reads from a single run. These reads were blasted against a repeat masked version of the macaque genome. Of the reads 42% exhibited a high match score with the macaque genome. It is likely that the remaining reads aligned to repeat masked regions of the genome or represent artifacts or DNA sequence from exogenous organisms in the original blood sample from which DNA was extracted. Of the total number of reads, 58% did not match the macaque genome with a ≥98% identity, 20% exhibited a perfect match with the DNA sequence from the macaque genome and 8% contained the information for candidate SNPs (Figure 1). After filtering, we identified 22,892 candidate SNPs from a single run (Table S1). The SNPs are evenly distributed across all chromosomes in the genome (Figure 2). However, the expected (based on simulations) and observed numbers of sequence overlaps were statistically significantly different from each other (p = 0.0001). The excess number of overlaps is likely due to an “inefficiency” in the 454 Life Science™ system resulting in beads with the same DNA segment being amplified and sequenced multiple times (454 Life Science™ pers com). This excess number of overlaps in the sequencing system is desirable for SNP identification and verification and as a result we were able to obtain at least 2×coverage on 1,947 SNPs. We trimmed this overlap SNP set by removing SNPs that exhibited a Sanger quality score below 60 in the draft sequence or a pyrosequencing score below 20. This resulted in an overlap set comprising 1,559 SNPs distributed across the genome (Figure 3). The transition/transversion ratio is within the expected limits (2.36) [6] and provides confidence that the SNPs in the overlap are not due to artifact or error. Previous studies using the same sequencing technology also report statistics consistent with a low level of error once the sequences have been appropriately screened for quality [7].

### Confirmation of Candidate SNPs

We were able to confirm the presence of 26 high-frequency SNPs (63.4%) out of the 41 loci sequenced in a pooled sample of four rhesus macaques (Table 1). Five of the 21 high-frequency SNPs were found in regions characterized as homologues to genes in humans. Based on the small number of individuals in the pooled sample the SNP frequencies should be considered as approximations. It is likely that most of the remaining 15 SNPs are of low-frequency but some may be useful as population specific markers for ancestry identification. A subset of three SNPs confirmed in the pooled sample was also confirmed with 100% accuracy in the DNA sequences of individuals used in the pooled sample. These results suggest that a majority of the candidate SNPs identified using this method will be high frequency and will be useful genetic markers for mapping QTLs, genetic management [8], and determining relatedness.

**Table 1:** Candidate SNPs confirmed through Sanger sequencing.

	Location	Pyrosequencing ID	SNP			
**1**	Chr02	D8YOWMI01DTGIR	M			
**2**	Chr03	D8YOWMI02GNQT9	M	A	C	N
**3**	**Chr03**	**D8YOWMI02GSRQO**	**M**	0.667	0.333	6
**4**	Chr04	D8YOWMI02HP0CJ	M			
**5**	Chr05	D8YOWMI01BHQA0	M			
**6**	Chr06	D8YOWMI02FG8XU	K			
**7**	Chr06	D8YOWMI01CG0QE	M			
**8**	Chr06	D8YOWMI01BSRVQ	S			
**9**	Chr06	D8YOWMI02IWMJX	M			
**10**	Chr07	D8YOWMI02J5FHH	K			
**11**	Chr07	D8YOWMI01A6K13	K			
**12**	Chr09	D8YOWMI02IVOYO	M			
**13**	Chr10	D8YOWMI02FZMH9	M			
**14**	Chr11	D8YOWMI02HKHPD	Y			
**15**	Chr13	D8YOWMI01ETU8L	K	G	C	N
**16**	**Chr15**	**D8YOWMI01E4TU3**	**S**	0.750	0.250	8
**17**	Chr15	D8YOWMI02F2CCM	S			
**18**	Chr16	D8YOWMI01BSPN1	R			
**19**	Chr17	D8YOWMI01BBO36	Y			
**20**	Chr17	D8YOWMI02JABAC	M			
**21**	Chr18	D8YOWMI01ADGFV	R			
**22**	Chr19	D8YOWMI01ELUBW	K	G	T	N
**23**	**Chr19**	**D8YOWMI02FS2LX**	**K**	0.750	0.250	8
**24**	ChrX	D8YOWMI02GK6I6	K			
**25**	ChrX	D8YOWMI01A5TTU	M			
**26**	ChrX	D8YOWMI01C7Z8G	S			

SNPs are labeled with the appropriate IUB ambiguity code. The SNPs that were confirmed in individual samples as well as the pooled sample are in bold. The major and minor allele frequencies are given. N = the number of chromosomes sampled.

## Discussion

### Experimental and Computational Information Integration

It is a non-trivial task to manage and analyze hundreds of thousands of sequences and their associate computational results, especially in a complete genome context. We created a database that has integrated 454 Life Science™ DNA sequences, their corresponding quality scores, BLAST outputs, rhesus macaque genome sequences and their corresponding Sanger quality scores. The database is built on top of MySQL with a web interface that allows dynamic queries through Perl CGI (Common Gateway Interface). The website for this database dubbed *Mamu*SNP, can be accessed at http://mamusnp.ucdavis.edu. *Mamu*SNP supports queries on one or more 454 sequence IDs to create a summary report with 454 sequences, relative positions of each base pair, quality scores in a table format and with mismatch sequences high-lighted by a different color. The details of BLAST outputs including BLAST scores and the sequence alignments with reference are also shown in the report. The query feature is a useful tool for verifying computational results and SNP candidate selection. Since queries concerning chromosome number and map positions are supported, this tool is particularly useful to search for novel SNPs in a particular genomic region of interest, for screening possible cases of overlaps between 454 sequences within a genomic region and for the retrieval of sequences flanking the target template. The latter facilitates primer design for the experimental verification of candidate SNPs.

## Materials and Methods

We chose to pyrosequence an animal from western China to maximize diversity when compared to the draft sequence from a rhesus macaque of Indian ancestry. Sample Sch00R1684 was obtained from Sichuan Province, China and the provenience of this sample was confirmed through mitochondrial DNA (mtDNA) sequencing (see Genbank Accession # DQ373245: [9]). This sample was chosen for analysis because there is a high probability, based on its nuclear (STR) and mitochondrial genome, that this animal has no admixture with eastern Chinese or Indian rhesus macaques in its ancestry [10]. Three separate DNA extractions were performed from *M. mulatta* blood sample Sch00R1684 using a QiaAmp Blood Extraction Mini Kit. The three extracts were pooled, purified, and concentrated using a Millipore Microcon YM-100 centrifugal Filter Device. Five micrograms of concentrated extract (at>300 ng/ul) was submitted to 454 Life Sciences in Branford, CT for pyrosequencing.

### Analysis and alignment of Raw Sequences

All DNA sequences once received from 454 were blasted against the MMUL 1.0 genome assembly (http://www.genome.wustl.edu/genome.cgi?GENOME = Macaca%20mulatta&SECTION = assemblies) from the Baylor College of Human Medicine. Prior to the search, repetitive sequences and low quality regions were masked out of the database at Baylor. The alignment was performed with NCBI's BLASTN variant of the BLASTALL program [11] without any low-complexity filters and an expectation value of 10.0. Chromosome, 454 sequence ID, alignment location, mismatch information, and gap information was parsed with a Perl script using BioPerl modules. To standardize conventions, alignments and mismatch locations were relabeled relative to the 5′ strand of the chromosome.

### Identification of SNP Candidates

To avoid reporting non-informative sequence polymorphisms, sequence alignments between 454 sequences and the reference genome that corresponded to variable MHC regions, and regions showing a statistically improbable number of sequence overlaps (>4 overlaps) were removed from further analysis. Only alignments with ≥98% identity or only one mismatch, with the second alignment scoring worst than the first were accepted for analysis. An identity match of 98% was selected to allow one or two mismatches on a single sequence. To control for pyrosequencing read errors in homopolymeric regions due to non-linear light response [12], any mismatch that was an extension/truncation of an existing homopolymeric repeat was removed from further analysis. All mismatches occurring within 5 bp of a gap were screened out. If overlapping alignments from 454 re-sequencing artifacts contain conflicting reports of mismatches the mismatch site was screened out. Any mismatch passing the aforementioned filters was considered as a potential SNP candidate.

### Confirmation of SNP Candidates

We screened a subset of candidate SNPs using Sanger sequencing of a pooled sample. The putative SNPs were first verified and checked for paralogous sequences by comparing the 454-generated fragment sequence to the rhesus macaque genome, using NCBI's rhesus macaque genome megaBLAST resource. (http://www.ncbi.nlm.nih.gov/projects/genome/seq/BlastGen/BlastGen.cgi?taxid = 9544). Fragments that matched multiple regions of the genome were discarded to avoid complications during primer-design and genotyping. Forty-one SNP loci in forty-one independent fragments were chosen for Sanger sequencing confirmation. After identifying the candidate loci, 400 bp of flanking sequence on either side of the SNP were retrieved using the ‘Sequence Query’ feature of the *Mamu*SNP web interface (http://mamusnp.ucdavis.edu); these sequences were used to generate oligonucleotide primers.

We created a pool of four *M. mulatta* samples exhibiting different mitochondrial DNA haplogroups and sequenced the forward and reverse strand of DNA fragments encompassing the candidate SNPs. By sequencing eight chromosomes in a pooled sample we were able to identify SNPs exhibited in the total population at greater than 12.5% in frequency. We define SNPs at this frequency or greater as “high-frequency SNPs.” The peaks of the electropherograms for the polymorphic site were visually inspected and if more than one peak was present at this position in both forward and reverse sequences it was considered to be a high-frequency SNP. Three loci that exhibited high-frequency SNPs in the pooled sample were randomly sequenced for all four individuals to confirm the SNP in individuals as well as the pooled sample. The frequencies of the major and minor alleles for these SNPs are given in Table 1.

## Supporting Information

Table S1 List of candidate SNPs.
(6.91 MB XLS)
